# Special Pathology in the course content of Third Year MBBS: Views of students and teachers

**DOI:** 10.12669/pjms.36.5.2397

**Published:** 2020

**Authors:** Riaz Shahbaz Janjua, Jamilah Riaz, Wafa Omer

**Affiliations:** 1 Dr. Riaz Shahbaz Janjua, HBS Medical & Dental College, Islamabad, Pakistan; 2 Dr. Jamilah Riaz Director Medical Education, HBS Medical & Dental College, Islamabad, Pakistan; 3 Dr. Wafa Omer Associate Professor of Pathology, HBS Medical & Dental College, Islamabad, Pakistan

**Keywords:** Curriculum, Students, Medical, Teachers

## Abstract

**Objective:**

The students and teachers are major stakeholders whenever there is a change in the curriculum. Objective of the study was to assess the views of Third Year MBBS students and college teachers involved in teaching Third Year MBBS class regarding the inclusion of special pathology to the already cumbersome course content.

**Methods:**

It was a cross-sectional descriptive study carried out over a period of eight months from April, 2019 to December, 2019. An online questionnaire was used to collect the data from 110 third year MBBS students and 35 medical college teachers involved in teaching the third year MBBS class at HBS Medical & Dental College, Islamabad, Pakistan. The questionnaire contained open ended questions along with a short questionnaire based on 3-point Likert scale for a semi-quantitative analysis. The open ended responses in the interviews were assessed using Mayring’s qualitative context analysis. The similar comments were bundled up as the comments were sequentially processed and the replicates were grouped. The responses were then ranked by the number of times they were selected using Microsoft Excel 2013 and SPSS 21.

**Results:**

A total of 105 medical students and 32 medical teachers participated in the study. n=94 (89.5%) of the students agreed that the content taught was incoherent and n= 92 (88%)agreed that the time allocation for the various modules was inappropriate. The important reservation of the students was that the assessment strategies of the past continued to prevail and they were not aligned with the change in the curriculum. They suggested to spread Pathology over four years of MBBS so that true integration can be done. The top ranked reason amongst the students who were in favor of this system was that they could easily leave microbiology on choice and study selectively to pass the exam as the extensive course inhibited the examiners to assess every aspect of Pathology comprehensively especially Microbiology and general Pathology being compromised upon. As far as the teachers are concerned n=28 (88%) agreed that the course content is inappropriate and the students are being bombarded with selective knowledge in a shorter period of time. Important reservation of the faculty members was that they were not trained to deliver the content according to this sudden change which has seriously affected the student’s results.

**Conclusions:**

Although curriculum change is a dynamic process and leads to refinement of the existing content but it should be implemented after proper planning, training and validation so that the students and the teachers can cope with and derive maximum benefit.

## INTRODUCTION

Curriculum revision is encouraged and is frequently practiced in the medical colleges of Pakistan.^1^ Over the past few years with the introduction of the integrated modular curriculum; the medical institutions in Pakistan are rigorously trying to modify the existing curriculum. However, the criteria and the duration after which these revisions should be made is still unclear.^2^ The students and teachers are major primary stakeholders whenever there is a change in the curriculum^3^ Their views and concerns are a significant variable in order to implement any change and a failure to do so deskills both students and teachers leading to failure in achieving the desired learning objectives. Curriculum revision and evaluation is a dynamic process but it needs to be implemented according to the rules of curricular organization and validation.^4^ Integrated modular curriculum was introduced in our setup with the intention of reducing the repetition of knowledge and for exposing the students to the academic load in a way that it is palatable and is free of unnecessary content.

The idea behind an integrated curriculum was to make the learning experience of the students more meaningful rather than bombarding them with excessive content and camouflaging our teacher centered beliefs of content delivery behind a veil of student centered activities. Recently a change in curriculum was abruptly introduced and Systemic Pathology which was taught earlier in 4^th^ year MBBS was shifted to 3^rd^ year MBBS along with General Pathology and Microbiology, Forensic Medicine and Pharmacology. Four medical Colleges namely Islamabad Medical and Dental College, Rawal Medical College, Federal Medical and Dental College and HBS Medical and Dental College affiliated with Shaheed Zulfiqar Ali Bhutto University endorsed the change. HBS Medical and Dental College, Rawal Medical College and Federal Medical and Dental College observed a drastic drop in the academic performance of the students during their internal block exams along with harsh feedback from the students and the senior faculty members.

The students and faculty members of Islamabad Medical and Dental College however seemed to be satisfied with the change though they did not share the details of their program and modified table of specifications with the rest of the stakeholders for critical appraisal (IMDC/2020/11 minutes of meeting, Dated 20-11-2020). Hence, objective of the study was to assess the views of students of 3^rd^ Year MBBS and the faculty of Pathology, Forensic Medicine and Pharmacology involved in teaching this class at HBS Medical and Dental College to understand the problems faced by these primary stakeholders so that we can come to a favorable solution for the problems encountered.

## METHODS

It was a cross-sectional descriptive study carried out over a period of eight months from April, 2019 to December, 2020. An online self-completion questionnaire was used to collect the data from 110 Third Year MBBS students and 35 medical college teachers involved in teaching the Third Year MBBS class at HBS Medical & Dental College, Islamabad, Pakistan. The study was approved by the Institutional Ethical Review Board (Ref. HBSMDC/2020/01, Dated: 29-01-2020) and informed consent of the study participants. Those who failed to give consent were excluded from the study. The questionnaire contained open ended questions along with a short questionnaire based on 3-point Likert scale (agree, neutral, disagree) assessing the views of students and teachers.^5^ The first segment of the questionnaire consisted of nine statements under the following major categories; time management and the appropriateness of content. The students and the faculty had to respond according to a 3-point Likert scale. The second segment consisted of 10 open ended questions. The participation was purely voluntary and the questionnaires were kept anonymous and confidential. See appendix.

### Statistical Analysis

The responses of the first segment were evaluated according to the 3-point Likert Scale and their frequency (percentages) calculated. The answers to open ended questions in the second segment were ranked keeping in view the number of times a particular response was given. The open ended responses in the interviews were assessed using Mayring’s qualitative context analysis (Herrmann et al.,2015). The similar comments were combined as the comments were sequentially processed and the replicates were grouped. The responses were then ranked by the number of times they were selected using Microsoft Excel 2013 and SPSS 21. The evaluators ranked the responses and discussed categorization. The interviews questionnaires were reviewed multiple times by the evaluators to reach a consensus.

## RESULTS

One hundred and five medical students of 3^rd^ year MBBS and 32 medical teachers of HBS Medical and Dental College who were actively involved in teaching the 3^rd^ year MBBS class participated in the study. Ninety two (88%) of the students and 23 (72%) of the teachers agreed that the time allocation for the various modules was inappropriate (Table-I).

**Table-I:** Student views regarding time management after the curriculum change.

Time Management	STUDENTS (n=105)		

	Agree n(%)	Neutral n (%)	Disagree n(%)
Was appropriate time assigned for the content covered?	10 (9.6)	3 (2.8)	92 (87.6)
Did the time efficiency improve after inclusion of Special Pathology to the course content?	12(11.5)	10(9.5)	83 (79)
Was the content covered in the given time coherent?	8 (7.7)	3 (2.8)	94 (89.5)
Did the time assigned focus more on quantity rather than quality?	92(87.6)	7 (6.7)	6 (5.7)
Was it easy for you to study 450 hours of Special Pathology along with Pharmacology and Forensic Medicine?	16(15.2)	13(12.3)	76 (72.4)

n= number of subjects; %=Percentage.

Detailed views of teachers regarding time management after this change in the curriculum content are shown in Table-II.

**Table-II:** Views of Teachers regarding time management after the curriculum change.

Time Management	TEACHERS (n=32)		

	Agree n (%)	Neutral n (%)	Disagree n (%)
Was appropriate time assigned for the content covered?	7 (21.8)	2 (6.3)	23 (71.8)
Did the time efficiency improve after inclusion of Special Pathology to the course content?	5 (16.8)	4 (13.9)	22 (69.3)
Was the content covered in the given time coherent?	8 (25)	2 (6.3)	22 (68.6)
Did the time assigned focus more on quantity rather than quality?	28 (87.5)	1 (3.1)	3 (9.4)
Were you able to teach 450 hours of Special Pathology along with Pharmacology and Forensic Medicine judiciously?	6 (18.7)	2 (6.3)	24 (75)

n= number of subjects; %=Percentage.

One of the major concerns was that only 25% of the students and 13% teachers thought that the current change in curriculum of Pathology would benefit them during their clerkship years ahead. As far as the teachers are concerned 80% agreed that the course content is inappropriate and the students are being bombarded with selective knowledge in a shorter period of time. The students were unable to apply the underlying principles of general pathology to special pathology as they were unable to understand the content of general pathology owing to the extensive course and shortage of time. The main problem that the students highlighted was that the learning objectives were vague and teachers and the course content was compromised as compared to the content delivered to the senior batch. ( Tables III & IV).

**Table-III:** Student views regarding appropriateness of content delivered.

Appropriateness of content delivered	STUDENTS (n=105)		

	Agree n(%)	Neutral n (%)	Disagree n(%)
Was only the ‘must know’ content delivered	91 (86.6)	5(4.8)	9(8.6)
Do you think knowledge was compromised upon	100 (95.2)	1(0.96)	4(3.8)
Was the content appropriate	48 (45.7)	2(1.9)	55 (52.3)
Do you think the content will benefit you during the clinical clerkships	26 (24.7)	8(7.6)	71(67.6)

n= number of subjects; %=Percentage.

**Table-IV:** Views of Teachers regarding appropriateness of content delivered.

Appropriateness of content delivered	TEACHERS (n=32)		

	Agree n (%)	Neutral n (%)	Disagree n (%)
Was only the ‘must know’ content delivered	26 (81)	3(9.4)	3(9.4)
Do you think knowledge was compromised upon	29 (90.6)	2(6.3)	1(3.1)
Was the content appropriate	13(40.6)	1(3.1)	18 (56.2)
Do you think the content will benefit the students during the clinical clerkships	4(12.5)	8(25)	20(62.5)

n= number of subjects; %=Percentage.

The foremost reservations of the students were that the assessment strategies of the past continued to prevail and they were not aligned with the change in the curriculum while that of the teachers was that they were not trained to deliver the content according to this sudden change and the results of the students had suffered considerably due to this change (Table-V).

**Table-V:** Participant reservations regarding various aspects of inclusion of Special Pathology in the 3^rd^ Year MBBS Course Content.

	Teachers (N=32) n (%)	Students (N=105) n (%)
Assessment was not congruent with the curriculum change	29 (73)	90 (76)
Learning objectives were not specific and uniform across the affiliated colleges.	27 (32)	88 (45)
Students and faculty were not informed about the proposed time of implementation	31 (95)	96 (63)
Lack of coordination between the affiliated colleges	25 (34)	79 (59)
Lack of faculty and student knowledge and preparation for the paradigm shift	29 (60)	92 (54)
Clinical training sessions were not uniform amongst the affiliated colleges.	30 (90)	90 (85)
No specific reservation	12 (19)	45 (18)

n= number of subjects; %=Percentage.

The foremost suggestion of the students and faculty was to spread Pathology over four years of MBBS so that true integration can be done (Table-VI).

**Table-VI:** Participant Recommendations for Future Sessions.

	Teachers (n=32) n (%)	Students (n=105) n (%)
Spread course content of Pathology over 4 years for better integration	25 (78)	97 (92)
Systemic Pathology has a lot of clinical relevance and should be made part of the clerkship years.	22 (69)	89 (85)
General and Special Pathology can be capped in third year with Pharmacology provided Forensic Medicine is moved to the clerkship years.	18 (56)	81 (77)
Lab Medicine is an integral part of Clinical Clerkship and rotation of students in the diagnostic laboratory should be ensured.	29(90)	95 (90)
Curriculum change should always be made after taking the faculty members and students into confidence as the major stakeholders.	31 (97)	99 (94)
Curriculum change should be made prospectively rather than retrospectively so that it does not disturb the sessions already in progress.	23 (72)	79 (75)
Curriculum change should be implemented after thorough external validation and should be evaluated annually.	25 (78)	81 (77)

n= number of subjects; %=Percentage.

## DISCUSSION

Curriculum change is a gradual process and its implementation requires extensive measures.^4^ This study shows that the sudden shift of Special Pathology to 3^rd^ Year MBBS along with General Pathology, Forensic Medicine and Pharmacology led to a number of problems highlighted by the faculty members and the students. Time management remains an essential component of successful delivery and understanding of the course content. According to the views analyzed the course content was not congruent with the time allotted due to which teachers had to stick to the ‘must know’ content which led to knowledge of the students being compromised.^6^

The students were bombarded with content by Forensic Medicine, Pharmacology and Pathology as the faculty had not been given a chance to prepare for this curriculum change neither were the students well prepared to modify their learning strategies to cope up with this abrupt change.^7^ One of the reasons for the students and the faculty to be unable to manage time effectively could be that they were not trained or equipped with the proper teaching and learning strategies before the change was implemented.^8^ The major reservation of the students and the teachers was that the assessment strategies were not congruent with the change in the curriculum^9^ and while the learning objectives across the different colleges were different the exam paper remained the same for all the affiliated colleges of SZABMU leading to deteriorating results shown by the students in the Block Exams. A possible reason for this might be the lack of coordination between the affiliated colleges and a communication gap between the students and faculty members of the affiliated colleges.^10^

Another observation was that one of the affiliated colleges was not sending the 3^rd^ Year class for clinical rotation unlike the remaining three colleges where 3^rd^ Year MBBS class was being sent for clinical rotation once a week. This might have been another contributing factor to the difference in the response of students to the change in curriculum. Clinical exposure since the very beginning of medical school has been encouraged and its importance has been highlighted in multiple studies^11^ therefore all the colleges should have been taken into confidence and this discrepancy should have been pointed out for better alignment of learning objectives. An important recommendation which came forward was that Pathology should be spread over four years of medical school and should not be capped in third year alone. A possible reason for this could be that the topics in General Pathology can be integrated very well with Anatomy, Physiology and Biochemistry.^12^ An example of this integration is that cell injury can be taught with the normal structure and function of the cell while Genetics can be integrated with the structure and function of DNA taught in Biochemistry. Special Pathology has two components Systemic Pathology and Laboratory Medicine or Diagnostic Pathology. So while Systemic Pathology can be taught in 3^rd^ Year with effective integration with Pharmacology and Forensic Medicine, Laboratory Medicine on the other hand can be made part of the clinical clerkship years to give a strong understanding to the student of the diagnostic pathology modalities discussed during multi-disciplinary team sessions.^13^,^14^

Students and faculty emphasized that laboratory medicine should be made part of the clinical clerkship years and that this aspect is largely neglected in the curricula of medical schools.^15^ This is in agreement with previous studies which state that laboratory medicine is essential for the student to correlate the clinical scenario with the diagnostic modalities and advise the investigations accordingly upon being conferred the degree of medicine.^16^ In a study by Ghanchi et al, medical students in Pakistan have previously also shown immense interest in the field of Pathology and its integration in the clinical years.^17^ Clinical relevance has been stressed upon by the students and faculty members in the previous studies as well.^18^

Last but not the least the recommendation unanimously given was that curriculum change should be implemented after proper planning, training and validation. The most probable reason for this recommendation was that the faculty and students both felt at a loss of teaching and learning strategies when the change was implemented abruptly.^19^ The strength of this study is that this is the only study to the best of our knowledge which has brought forward the views of the primary stake holders after this change was introduced in the affiliated colleges of SZABMU.

### Limitations of this study

Apart from the primary stake holders who are the students and the teachers there are a number of other stake holders too like the curriculum committee members, QEC and institutional administrators who could not be made part of this study due to time and resource constraints. We intend to broaden the scope of this study in future and document the views of other stake holders as well.

## CONCLUSIONS

Although curriculum change is a dynamic process and leads to refinement of the existing content but it should be implemented after proper planning, training and validation so that the students and the teachers can cope with the change and derive utmost benefit from it.

### Authors’ Contribution

**RSJ, JR & WO:** Conceived the idea and designed the study, Collected the data are responsible for integrity of research.

**RSJ & WO:** were involved in the data analysis and data interpretation.

All the authors contributed towards writing the manuscript and they approved the final version.

## ANNEXURE-1

**QUESTIONAIRRE STUDENTS**

**Table:**

Please tick one option 1=Agree, 2=Neutral, 3= disagree				

Name:	Roll Number:	Students Form		
***Time Management***		1	2	3
a. Was appropriate time assigned for the content covered?				
b. Did the time efficiency improve after inclusion of Special Pathology to the course content?				
c. Was the content covered in the given time coherent?				
d. Did the time assigned focus more on quantity rather than quality?				
e.Was it easy for you to study 450 hours of Special Pathology along with Pharmacology and Forensic Medicine?				
***Appropriateness of content***			
f. Was only the ‘must know’ content delivered				
g. Do you think knowledge was compromised upon				
h. Was the content appropriate				
i. Do you think the content will benefit you during the clinical clerkships.				

**Please tick one option 1=Agree, 2=Neutral, 3= disagree**				

Name:	Designation:	Teachers Form		

***Time Management***		1	2	3

a. Was appropriate time assigned for the content covered?				
b. Did the time efficiency improve after inclusion of Special Pathology to the course content?				
c. Was the content covered in the given time coherent?				
d. Did the time assigned focus more on quantity rather than quality?				
e. Were you able to teach 450 hours of Special Pathology along with Pharmacology and Forensic Medicine judiciously?				
***Appropriateness of content***				
f. Was only the ‘must know’ content delivered				
g. Do you think knowledge was compromised upon				
h. Was the content appropriate				
i. Do you think the content will benefit the students during the clinical clerkships				

**SEGMENT-2: OPEN ENDED QUESTIONS FOR STUDENTS AND TEACHERS**				

***Participant Reservations***
1. What are your reservations regarding the assessment strategies?
2. What are your reservations concerning the learning objectives of the newly implemented course?
3. What reservation do you have regarding its implementation?
4. What reservations do you have regarding the clinical training sessions?
5. Please mention if you have no specific reservation
***Participant Recommendations for Future Sessions.***
6. What changes do you recommend regarding the course distribution of Pathology?
7. What are your recommendations regarding the significance of Pathology in the clerkship years?
8. How do you think Pathology can be capped in third year?
9. What are your views and recommendations keeping in view the implementation of this curriculum change during the session?
10. How can we improve upon this change?

Thank you for your participation.
